# Association of Discontinuing Preinjury Beta-Adrenergic Blockade Medications With Mortality in Severe Blunt Traumatic Brian Injury

**DOI:** 10.1097/AS9.0000000000000324

**Published:** 2023-08-29

**Authors:** Christopher J. Tignanelli, Saman Arbabi, Gaby Iskander, Kurt Kralovich, John Scott, Naveen F. Sangji, Mark R. Hemmila

**Affiliations:** From the * Department of Surgery, University of Minnesota, Minneapolis, MN; † Department of Surgery, University of Washington, Seattle, WA; ‡ Division of Acute Care Surgery, Spectrum Health, Grand Rapids, MI; § Department of Surgery, Henry Ford Hospital, Detroit, MI; ‖ Department of Surgery, University of Michigan, Ann Arbor, MI.

**Keywords:** traumatic brain injury, beta blocker, trauma, health services research, trauma outcomes

## Abstract

**Background:**

Beta-adrenergic receptor blocker (BB) administration has been shown to improve survival after traumatic brain injury (TBI). However, studies to date that observe a benefit did not distinguish between continuation of preinjury BB versus de novo initiation of BB.

**Objectives:**

To determine the effect of continuation of preinjury BB and de novo initiation of BB on risk-adjusted mortality and complications for patients with TBI.

**Methods:**

Trauma quality collaborative data (2016–2021) were analyzed. Patients were excluded with hospitalization <48 hours, direct admission, or penetrating injury. Severe TBI was identified as a head abbreviated injury scale (AIS) value of 3 to 5. Patients were placed into 4 groups based on the preinjury BB use and administration of BB during hospitalization. Propensity score matching was used to create 1:1 matched cohorts of patients for comparisons. Odd ratios of mortality accounting for hospital clustering were calculated. A sensitivity analysis was performed excluding patients with AIS >2 injuries in all other body regions to create a cohort of isolated TBI patients.

**Results:**

A total of 15,153 patients treated at 35 trauma centers were available for analysis. Patients were divided into 4 cohort groupings related to preinjury BB use and postinjury receipt of BB. The odds of mortality was significantly reduced for patients with a TBI on a preinjury BB who had the medication continued in the acute setting (as compared with patients on preinjury BB who did not) (odds ratio [OR], 0.73; 95% confidence interval [CI], 0.54–0.98; *P* = 0.04). Patients with a TBI who were not on preinjury BB did not benefit from de novo initiation of BB with regard to mortality (OR, 0.83; 95% CI, 0.64–1.08; *P* = 0.2). In the sensitivity analysis, excluding polytrauma patients, patients on preinjury BB who had BB continued had a reduction in mortality when compared with patients in which BB was stopped following a TBI (OR, 0.65; 95% CI, 0.47–0.91; *P* = 0.01).

**Conclusions:**

Continuing BB is associated with reduced odds of mortality in patients with a TBI on preinjury BB. We were unable to demonstrate benefit from instituting beta blockade in patients who are not on a BB preinjury.

## Abstract 2

Mini Abstract:

Although prior studies suggest a benefit for the initiation of beta-adrenergic receptor blockers (BB) in the treatment for traumatic brain injury (TBI), none characterized the difference between continuation of preinjury BB versus de novo initiation of postinjury BB. This study of 35 ACS-verified trauma centers is the first to determine the effect of preinjury and postinjury BB usage on risk-adjusted mortality and complications in TBI patients. For patients on preinjury BB, we found that discontinuing BB in the setting of a TBI is associated with an increased risk of mortality and complications. In patients not already on a BB preinjury, there may be no benefit to initiation of BB therapy in the setting of a TBI with regard to mortality.

## INTRODUCTION

Observational studies, meta-analyses, and a recent prospective randomized clinical trial have demonstrated improved outcomes for traumatic brain injury (TBI) patients receiving in-hospital treatment with beta-adrenergic receptor blockers (BB).^1–5^ Previous studies of BB therapy have not accounted for preinjury BB medication use in TBI patients. There are 2 relevant clinical scenarios in TBI patients: (1) continuation of preinjury BB (preinjury BB use and received versus did not received in-hospital BB use) and (2) de novo initiation of BB (no preinjury BB use and received versus did not received in-hospital BB use).

Few studies to date in TBI captured patient preinjury BB use and thus it is possible the beneficial effect of postinjury BB initiation observed in the literature is related to continuation of preinjury BB rather than de novo BB initiation. This hypothesis aligns with literature regarding BB continuation in the perioperative setting, which has been found to reduce postoperative complications nearly twofold and represents the current standard of care.^6–8^ To investigate this hypothesis, we used prospectively collected data regarding preinjury BB use and in-hospital TBI BB use from the Michigan Trauma Quality Improvement Program (MTQIP). The MTQIP is a collaborative quality initiative focused on performance improvement (PI) in level I and II trauma centers.^9,10^ Thus, the objective of this study was to investigate the effect of continuation of preinjury BB and de novo initiation of BB on risk-adjusted mortality and complications for patients with TBI.

## METHODS

### Data Collection

A retrospective cohort study design was conducted using data collected from January 1, 2016 to June 30, 2021, of 35 US trauma centers participating in the MTQIP, which is composed of all 35 American College of Surgeons Committee on Trauma verified level I and II trauma centers in Michigan.^11,12^ The MTQIP continuous quality improvement provides comprehensive risk-adjusted benchmark reports to participants in paper form and on a query-enabled website. Face-to-face collaborative meetings among participants are held 3 times per year. Trauma centers participating in the MTQIP conduct global PI projects and collect data related to these PI efforts (eg, venous thromboembolism prophylaxis, hemorrhage control and blood product utilization, and brain injury management).^13–15^ Data collection relies on the existing trauma registry and an add-on module inserted for collection of MTQIP specific data elements at participating hospitals. MTQIP uses a data definitions dictionary, based on the National Trauma Data Standard, which is published online and updated annually.^16,17^ Trauma registrars and data abstractors from the participating centers undergo annual training in MTQIP and National Trauma Data Standard data definitions. To ensure data fidelity, in addition to data standardization, MTQIP employs a program of robust data validation with feedback on an annual basis.^18^ All injury severity score (ISS) values were derived from registrar abstracted and recorded AIS 2005 codes with 2008 updates. Data are transmitted to the coordinating center at 2-month intervals.

### Study Population

**FIGURE 1.**
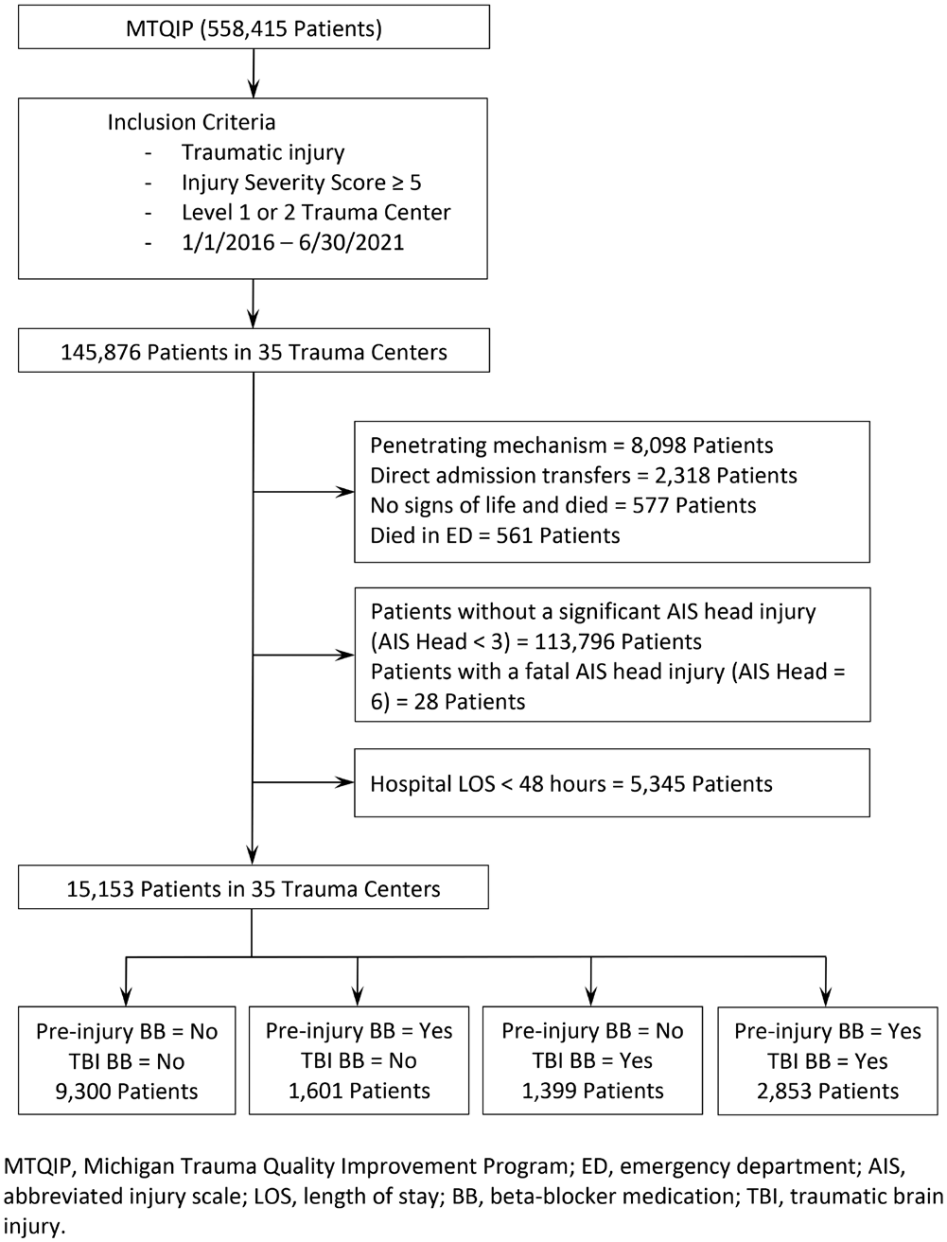
Flow chart for patient selection. AIS indicates abbreviated injury scale; BB, beta-blocker medication; ED, emergency department; LOS, length of stay; MTQIP, Michigan Trauma Quality Improvement Program; TBI, traumatic brain injury.

We used MTQIP data for patients admitted between January 1, 2016 and June 30, 2021. Patients were excluded if they were <16 years old, had an ISS <5, or did not have at least 1 diagnosis consistent with a traumatic injury (Fig. 1).^16^ Additional exclusion criteria included penetrating mechanism of injury, transfer out of the existing hospital emergency department (ED) to another hospital for definitive care, direct admission transfer in, evidence of no signs of life in the ED (ED systolic blood pressure = 0, ED heart rate = 0, and ED Glasgow Coma Scale (GCS) score = 3),^17^ or died in the ED. A significant head injury was defined as a head abbreviated injury scale (AIS) ≥3. Patients with a fatal AIS head injury (AIS head = 6) were excluded. Patients with a hospital length of stay <48 hours were also excluded. Preinjury BB usage was recorded as positive if the patient reported taking BB medication within a 2-week period before injury.

### Exposure

The primary exposure was the delivery of postinjury BB. Patients were considered to have received postinjury BB treatment if BB medication was administered within 48 hours after admission to the hospital. Based on the answers to preinjury BB use and in-hospital TBI treatment with BB, patients were placed in 1 of the 4 cohort categories: (−) preinjury BB use/(−) postinjury BB use, (+) pre-injury BB use/(−) postinjury BB use, (−) pre-injury BB use/(+) postinjury BB use, and (+) preinjury BB use/(+) postinjury BB use. These cohort groupings were used to compare preinjury and/or postinjury BB medication use as the exposure to patients not on BB medication before or after TBI.

### Outcome Variables

Mortality and hospice mortality or discharge were the primary outcomes assessed. Mortality was defined as a hospital disposition of death. The hospice mortality or discharge outcome is a composite measure signifying an in-hospital death or hospital disposition of discharge to hospice.^19^ Secondary outcomes evaluated included any complications, serious complications, cardiac complications (composite of cardiac arrest, myocardial infarction [MI], or cerebral vascular accident), cardiac arrest, MI, and cerebral vascular accident. Serious complications were defined as complications with an associated significant mortality rate within the MTQIP and included acute respiratory distress syndrome, acute renal failure, cardiac arrest, Clostridium difficile colitis, decubitus ulcer, deep vein thrombosis, enterocutaneous fistula, extremity compartment syndrome, MI, pneumonia (including ventilator-associated pneumonia), pulmonary embolism, renal insufficiency, stroke/cerebrovascular accident, systemic sepsis, unplanned admission to intensive care unit, unplanned intubation, and unplanned return to operating room.^12,13^

### Statistical Analysis

Univariate differences in patient characteristics by group were evaluated using χ^2^ tests for categorical variables and analysis of variance F tests for continuous variables. Unadjusted rates of mortality and hospice mortality or discharge were compared across BB cohorts using χ^2^ tests. Comparisons were made for the outcome of interest as the dependent variable with BB cohort as the independent variable.

A propensity score matching approach was used to create 2 evenly matched groups to evaluate whether preinjury and/or postinjury BB medication use was associated with improved outcomes compared with nonusage of BB medication. Specifically, 2 propensity matched cohorts were generated.

1. The first investigated de novo initiation of BB by comparing patients that were not on preinjury BB and did (versus did not) receive postinjury BB.
2. The second investigated continuation of preinjury BB, by comparing patients that were on preinjury BB and did (versus did not) receive postinjury BB.

To generate the propensity scores, for each comparison, we fit a logistic regression algorithm including relevant covariates. Relevant covariates considered for inclusion in the propensity model matching process were patient demographics (sex, race, age, and insurance status), injury mechanism, injury type and burden, patient physiology (initial ED systolic blood pressure, initial ED pulse rate, ED GCS motor score, and pupillary response), transfer status, and patient comorbidities. Additional TBI patient-specific findings and interventions were also included (midline shift, brain operation, intracranial pressure monitor use, timeliness operation or ICP monitor, and intubation status). Patient comorbid conditions were selected for inclusion in the model if they were identified to be significantly different between the cohorts based on the univariate analysis (*P* < 0.05). In some instances, specific incidents had missing values for potentially important covariates (eg, ED GCS motor score, ED systolic blood pressure, and ED pulse rate). To minimize bias, these values were categorized and accounted for using indicator variables. Injury burden was defined using a categorical variable for ISS with the following 4 categories (ISS: 5–15, 16–24, 25–35, and >35). To account for the differential effect of injury severity by injury body region, indicator variables for AIS score >2 in each of the face, extremity, chest, and abdominal regions were created and included.

All the covariates described above were entered into a logistic regression analysis, and a maximum-likelihood probit model was fitted based on these covariates as predictors of preinjury and/or postinjury BB medication use. The probit coefficients for these predictors were used to calculate a propensity score of 0 to 1 for each patient. Based on the calculated propensity scores, evenly matched groups were formed for each of the comparison groupings of BB usage with the common caliper set at 0.1. A univariate analysis was performed for each covariate to ensure there were no significant differences between covariates in each of the 2 propensity matched groups, see example Table S1, http://links.lww.com/AOSO/A237. Differences in outcomes for the cohorts of interest were explored using logistic regression both with and without accounting for clustering at the hospital level. A sensitivity analysis was performed excluding patients with AIS >2 injuries in all other body regions except the head to create a cohort of isolated TBI patients.

Results were expressed as unadjusted rates or adjusted odds ratios (ORs) for the outcomes of interest. The 95% confidence intervals (95% CIs) were calculated using variance estimates that account for patient clustering within hospitals. A *P* value <0.05 was used as the threshold for statistical significance. All analyses were performed in Stata MP 15.1 (StataCorp, College Station, TX). This study was submitted to the University of Michigan Medical School Institutional Review Board and given a determination of “not regulated” status as secondary use of data from a quality assurance and quality improvement clinical activity.

## RESULTS

A total of 15,153 patients treated at 35 trauma centers were available for analysis. Patients were divided into the following cohort groupings: Preinjury BB−/TBI BB− (N = 9300), pre-injury BB+/TBI BB− (N = 1601), pre-injury BB−/TBI BB+ (N = 1399), and pre-injury BB+/TBI BB+ (N = 2853). Significant differences were noted among the 4 unmatched groups for nearly all of the patient characteristics listed (Table S2, http://links.lww.com/AOSO/A238). Specifically for unmatched groups, patients on preinjury BB were more likely to be >75 years (57.2% and 62.0% for the 2 preinjury BB+ groups vs 27.4% and 37.8%; *P* < 0.001). Patients on preinjury BB were less likely to be intubated (37.5% and 27.8% vs 45.7% and 48.2%; *P* < 0.001) and less likely to receive an ICP monitor (4.8% and 2.4% vs 10.7% and 12.2%; *P* < 0.001).

### Analysis of Patients on Preinjury BB

Patients on preinjury BB that did not have BB continued after TBI diagnosis had a higher mortality than patients that had BB continued after TBI diagnosis (13.2% vs 6.8%; *P* < 0.001) for the unmatched cohort (Table S3, http://links.lww.com/AOSO/A239). This finding persisted when accounting for mortality or hospice for the unmatched cohort (21.1% vs 12.5%; *P* < 0.001). A sensitivity analysis was also conducted in patients with isolated TBI. Similar findings were observed, patients that did not have preinjury BB continued had higher mortality than patients with preinjury BB continued for the unmatched cohort (12.6% vs 6.6%; *P* < 0.001) (Table S3, http://links.lww.com/AOSO/A239).

Given possible selection bias for continuation of preinjury BB, a propensity-matched analysis was conducted. Following propensity matching, patients that did not have preinjury BB continued had increased mortality versus patients that did have preinjury BB continued (11.8% vs 8.9%; OR, 0.73; 95% CI, 0.54–0.98; *P* = 0.04) (Table S4A, http://links.lww.com/AOSO/A240). Patients that had a preinjury BB continued (as compared with patients that did not have preinjury BB continued) did not experience a significant increase in complications. A similar effect was observed for mortality in a sensitivity analysis of propensity-matched patients with isolated TBI if preinjury BB was continued (11.1% vs 7.5%; OR, 0.65; 95% CI, 0.47–0.91; *P* = 0.01) (Table S5A, http://links.lww.com/AOSO/A241).

### Analysis of Patients not on Preinjury BB

Patients not on preinjury BB that had de novo BB initiated after TBI diagnosis had a similar mortality as patients that had de novo BB initiated after TBI diagnosis (8.3% vs 7.8%; *P* = 0.5) for the unmatched cohort (Table S3, http://links.lww.com/AOSO/A239). A sensitivity analysis was also conducted in patients with isolated TBI. Similar findings were observed in patients with isolated TBI that had de novo (vs did not) BB initiated (7.2% vs 6.3%; *P* = 0.2) for the unmatched cohort (Table S3, http://links.lww.com/AOSO/A239).

In propensity-matched analysis of patients not on preinjury BB, we did not observe a significant difference in mortality between patients that did not have BB initiated versus those that did (9.7% vs 8.2%; OR, 0.83; 95% CI, 0.64–1.08; *P* = 0.2) (Table S4B, http://links.lww.com/AOSO/A240). Patients that were started on a BB who had not been on preinjury BB experienced significantly higher rates of many complications: any complication (29.5% vs 22.9%; OR, 1.41; 95% CI, 1.1–1.8; *P* = 0.005) and serious complications (25% vs 19%; OR, 1.73; 95% CI, 1.1–1.8; *P* = 0.002). A sensitivity analysis of patients with isolated TBI did not demonstrate a significant difference in mortality between propensity-matched patients that did have BB initiated versus those that did not (7.0% vs 8.8%; OR, 0.79; 95% CI, 0.59–1.04; *P* = 0.09) (Table S5B, http://links.lww.com/AOSO/A241). Despite no difference in mortality, patients that had BB initiated had significantly more complications versus those that did not: any complication (25.1% vs 19%; OR, 1.43; 95% CI, 1.1–1.8; *P* = 0.004) and serious complications (20.2% vs 14.5%; OR, 1.5; 95% CI, 1.1–2; *P* = 0.005).

## DISCUSSION

This study sought to determine the effect of de novo initiation of BB postinjury and continuation of preinjury BB following TBI. In patients not already on a BB preinjury, there may be no benefit to initiation of BB therapy in the setting of a TBI with regard to mortality. We observed that when a BB is started in TBI patients not on preinjury BB, these patients were at increased risk for complications. This, however, presents a chicken-or-the-egg causality dilemma, which we are unable to differentiate in this retrospective analysis as we lack detail regarding the temporality of BB initiation to complications. De novo BB are typically initiated in response to a complication, such as tachyarrhythmia, hypertension, myocardial ischemia, or neurostorming. Thus, de novo BB initiation may be an indicator of patients receiving treatment for more severe complicated disease. On the contrary, previously published observational studies and meta-analyses have identified a clinical benefit to BB treatment after TBI diagnosis; however, few have accounted for preinjury BB use and thus were unable to differentiate between de novo BB initiation versus BB continuation after TBI diagnosis. It is possible the beneficial effect observed in previous studies could be driven by continuation of BB rather than de novo initiation. Ultimately, to better infer causality, a multicenter randomized interventional trial is needed.

Our study has 2 key findings. First, we identified a significant 27% to 35% reduction in mortality for patients that had preinjury BB continued after TBI diagnosis. Although there are no studies to date regarding BB discontinuation in TBI, the effects of BB discontinuation have been studied since the 1970s especially in the postoperative setting.^6–8^ From the pathophysiologic standpoint, abrupt withdrawal of BB is associated with discontinuation syndrome, which occurs in 43% of patients.^20^ This is characterized by rebound hypertension and tachycardia, which predisposes patients to coronary events (35%), sudden death (5%), and cardiac arrhythmias (2%). Of note, we did not observe a significant increase in coronary events in TBI patients whom preinjury BB were discontinued post TBI. Specific to TBI patients, BB withdrawal syndrome may exacerbate secondary injury due to elevations in ICP. The prevention of BB discontinuation syndrome may explain the importance of BB continuation in other specialties where such practice is standard of care. For example, BB continuation is currently standard of care and a measured quality metric in the Surgical Care Improvement Project following noncardiac elective surgery.^21,22^ BB discontinuation following noncardiac elective surgery is associated with a twofold increase in adverse events, and this effect is significantly greater (sixfold) in patients with pre-existing cardiac risk.^23^

Second, we did not identify a benefit to de novo initiation of BB for patients with TBI. As discussed previously, our study is unable to infer temporality and it is possible that de novo BB initiation is rather a marker of more severe disease progression. Our finding conflicts with previously published observational studies and meta-analyses of those observational studies. Three previous meta-analyses of largely of the same observational studies identified a significant reduction in mortality in patients treated with BBs. Specifically, Chen et al’s meta-analysis included 13 observational studies of 15,734 patients and observed a 60% reduction in mortality (OR, 0.4; 95% CI, 0.3–0.54; *P* < 0.001).^24^ Alali et al’s meta-analysis included 9 observational studies of 6240 patients and observed a 61% reduction in mortality (OR, 0.39; 95% CI, 0.27–0.56; *P* < 0.001).^25^ Ding et al’s meta-analysis included 15 studies (12 observational and 3 randomized controlled trials) of 12,721 patients and observed a 61% reduction in mortality (OR, 0.39; 95% CI, 0.3–0.51]; *P* < 0.001).^4^ Of note, Ding further investigated the incidence of cardiopulmonary adverse events of 5 studies in patients that received BB exposure after TBI diagnosis (OR, 0.91; 95% CI, 0.55–1.5; *P* = 0.7). This finding of unchanged cardiopulmonary adverse events following post TBI BB treatment also contradicts our study’s findings. We observed a 73% increase (2% vs 3.3%; OR, 1.73; *P* = 0.02) in cardiac complications following de novo BB initiation. Although promising, these meta-analyses are limited by the inclusion of observational studies that were unable to differentiate between de novo versus continuation of beta-blockade. The largest randomized clinical trial was conducted in 2020 (219 patients that were not on preinjury BB) and investigated propranolol in patients with severe (AIS > 2) TBI.^5^ They did not identify a significant reduction in mortality for all patients with TBI (BB+ 8.1% vs BB− 16.7%; *P* = 0.058). However, given the effect size, it is possible a larger sample size may have identified a significant benefit supporting de novo BB initiation in patients with TBI. A subgroup analysis of 154 patients with isolated TBI did identify a significant reduction for in-hospital mortality (BB+ 4.4% vs BB− 18.6%; *P* = 0.012).

This study has multiple limitations to acknowledge. First, the study is observational in nature and not a clinical trial. Second, BB may have been discontinued due to the need to maintain cerebral perfusion pressure. Thus, BB discontinuation or noninitiation may serve as a proxy for injury severity. Similarly, BB initiation may be due to complications and thus a marker of more severe disease. Third, insufficient data are available to differentiate between the timing to initiate, type, dosage, held doses, and frequency of BB therapy. Lipophilic beta-blockers such as propranolol and metoprolol can cross the blood-brain barrier and directly activate adrenoreceptors on neuronal cells unlike hydrophilic beta-blockers such as atenolol. Fourth, the MTQIP database only prospectively captures if a patient received BB within 48 hours of admission, thus the effect of late or time to administration of BB is unable to be determined. Fourth, data are not present related to functional outcomes or long-term outcomes beyond hospital discharge. The strength of this study is that all data were intentionally collected and both locally and centrally validated by MTQIP thus enhancing data credibility.

This study offers an important caveat to previously published observational studies and meta-analysis of BB in TBI patients. Our findings suggest the positive effect observed from BB treatment in TBI may be conferred due to the continuation BB rather than de novo initiation of BB. Randomized controlled trials are needed to specifically evaluate and differentiate between the continuation of verses de-novo initiation of BB in patients with TBI. While there are 2 currently enrolling trials of BB in TBI, only 1 excludes patients who are on pre-existing BB.^26–28^ There are no currently enrolling studies to investigate continuation of BB in patients with a new TBI diagnosis.

## CONCLUSIONS

Continuing BB is associated with reduced odds of mortality in patients with a TBI on preinjury BB. We were unable to demonstrate benefit from instituting beta blockade in patients who are not on a BB preinjury.

## Supplementary Material


