# Supplementary material for: Association of Discontinuing Preinjury Beta-Adrenergic Blockade Medications With Mortality in Severe Blunt Traumatic Brian Injury
Source: Ann Surg Open. 2023 Aug 29;4(3):e324. doi: 10.1097/AS9.0000000000000324 (PMC10513140; doi:10.1097/AS9.0000000000000324)
Supplement: Supplementary file 1 [file as9-4-e324-s001.pdf]

Supplemental Table 1. Evaluation for Significant Differences in Patient Characteristics – After Propensity Match

A. De novo initiation of beta-blockers for patients with traumatic brain injury

Patient Characteristic	Cohort		*p*-value
	Pre BB = No	Pre BB = No	
	TBI BB = No	TBI BB = Yes	
Patients, N	1,387	1,387	
Age, mean (SD)	66.2 (19.5)	65.6 (19.8)	0.4
Age, %			
16-25y	3.8	4.4	0.8
26-45y	12.0	12.3	
46-65y	26.8	26.6	
66-75y	18.0	19.0	
>75y	39.4	37.7	
Male, %	62.6	62.8	0.9
Race, %			
White	76.9	76.9	0.5
Black	19.8	19.1	
Other	3.3	4.0	
Payment			
Commercial	30.3	30.7	0.7
Medicaid	9.8	10.5	
Medicare	51.8	51.8	
Uninsured/Self pay	5.3	4.2	
Other	2.8	2.8	
Injury, %			
Fall	72.2	71.4	0.98

MVC	10.6	11.4	
Motorcycle	4.2	4.4	
Pedal	5.1	5.2	
Struck	4.4	4.5	
Other	3.5	3.1	
Injury Severity Score, %			
5-15	33.0	30.9	0.6
16-24	30.6	31.6	
25-35	30.6	31.9	
>35	5.8	5.6	
AIS Head/neck>2, %	100.0	100.0	0.99
AIS Chest>2, %	13.4	13.6	0.9
AIS Abdomen>2, %	2.6	2.7	0.8
AIS Extremity>2, %	6.7	6.6	0.9
ED Heart Rate, %			
51-120, bpm	87.6	87.7	0.9
> 120	8.9	8.3	
0-50	1.3	1.4	
Missing	2.2	2.6	
ED Systolic Blood Pressure, %			
> 90, mmHg	94.7	94.7	0.8
61-90	2.2	1.9	
≤ 60	0.1	0.1	
Missing	3.0	3.3	
Glasgow Coma Scale Motor, %			
6	64.6	64.4	0.99
5-2	16.5	16.3	
1	11.8	11.9	

Missing	7.1	7.4	
Pupil Response			
Both Reactive	76.6	76.3	0.96
One Reactive	3.4	3.3	
Neither Reactive	5.3	5.1	
Unable to obtain or missing	14.7	15.3	
Midline Shift			
Yes	19.2	19.8	0.7
No	76.4	75.0	
Not imaged	0.5	0.7	
Missing	3.9	4.5	
Transfer in, %	21.8	23.0	0.4
Intubated, %	46.4	48.0	0.4
Pre-arrival CPR	1.1	1.0	0.9
ICP Monitor	13.1	12.1	0.4
Brain Operation	21.2	21.6	0.8
Time to Intervention < 8hrs	18.1	16.4	0.2
Comorbid diseases, %			
Active chemotherapy	0.6	0.8	0.5
Advanced directive	5.8	5.7	0.9
Alcohol use disorder	16.2	16.8	0.6
Angina	0.3	0.7	0.2
Bleeding risk	20.8	21.9	0.5
Cerebrovascular accident	5.3	4.8	0.5
COPD	7.6	8.2	0.6
Chronic renal failure	1.9	2.0	0.8
Congestive heart failure	5.8	6.4	0.5
Current smoker	19.9	21.1	0.4

Dementia	12.0	11.4	0.6
Diabetes mellitus	18.2	19.0	0.6
Disseminated cancer	0.9	0.9	0.99
Drug use disorder	13.2	14.5	0.3
Functionally dependent health status	20.8	21.2	0.8
History of myocardial infarction	0.6	0.9	0.4
Hypertension requiring medication	51.1	52.3	0.5
Liver disease	0.9	0.9	0.8
Major psychiatric illness	21.4	23.3	0.2
Obesity	2.9	2.7	0.8
Peripheral vascular disease	1.6	2.5	0.1
Steroid use	2.0	2.0	0.99

BB, beta blocker medication; TBI, traumatic brain injury; SD, standard deviation; y, year; MVC, motor vehicle crash; AIS, Abbreviated Injury Scale; ED, emergency department; bpm, beats per minute; mmHg, millimeters of mercury; ICP intracranial pressure monitor; hrs, hours; COPD, chronic obstructive pulmonary disease.

B. Continuation of pre-injury beta-blockers for patients with traumatic brain injury

Patient Characteristic	Cohort		*p*-value
	Pre BB = Yes	Pre BB = Yes	
	TBI BB = No	TBI BB = Yes	
Patients, N	1,502	1,502	
Age, mean (SD)	76.2 (12.7)	75.5 (13.0)	0.2
Age, %			
16-25y	0.0	0.1	0.5
26-45y	1.9	2.4	
46-65y	17.6	19.1	
66-75y	21.1	21.3	
>75y	59.4	57.1	
Male, %	56.7	57.3	0.7
Race, %			
White	84.9	84.4	0.9
Black	11.6	12.0	
Other	3.5	3.6	
Payment			
Commercial	16.4	19.4	0.04
Medicaid	4.7	4.9	
Medicare	75.0	70.3	
Uninsured/Self pay	2.6	3.5	
Other	1.3	1.9	
Injury, %			
Fall	86.9	85.3	0.8
MVC	7.0	8.2	
Motorcycle	1.3	1.3	

Pedal	1.3	1.7	
Struck	2.4	2.5	
Other	1.1	1.0	
Injury Severity Score, %			
5-15	37.3	36.4	0.9
16-24	33.6	34.0	
25-35	27.6	27.8	
>35	1.5	1.8	
AIS Head/neck>2, %	100.0	100.0	0.99
AIS Chest>2, %	7.0	8.0	0.3
AIS Abdomen>2, %	0.9	1.1	0.6
AIS Extremity>2, %	4.1	4.2	0.9
ED Heart Rate, %			
51-120, bpm	91.0	91.3	0.96
> 120	4.2	3.9	
0-50	1.3	1.4	
Missing	3.5	3.4	
ED Systolic Blood Pressure, %			
> 90, mmHg	94.4	94.5	0.99
61-90	1.7	1.7	
≤ 60	0.2	0.2	
Missing	3.7	3.6	
Glasgow Coma Scale Motor, %			
6	76.0	75.4	0.9
5-2	10.9	11.9	
1	4.1	4.1	
Missing	9.0	8.6	
Pupil Response			

Both Reactive	77.6	77.9	0.99
One Reactive	2.9	2.9	
Neither Reactive	2.4	2.4	
Unable to obtain or missing	17.1	16.8	
Midline Shift			
Yes	17.0	17.3	0.99
No	80.4	80.1	
Not imaged	1.1	1.1	
Missing	1.5	1.5	
Transfer in, %	29.0	29.4	0.8
Intubated, %	34.3	36.2	0.3
Pre-arrival CPR	0.5	0.4	0.8
ICP Monitor	3.3	3.7	0.6
Brain Operation	13.4	13.7	0.8
Time to Intervention < 8hrs	7.5	7.8	0.8
Comorbid diseases, %			
Active chemotherapy	1.0	0.9	0.9
Advanced directive	12.3	12.5	0.9
Alcohol use disorder	10.1	11.1	0.3
Angina	2.0	1.9	0.9
Bleeding risk	45.9	44.7	0.5
Cerebrovascular accident	8.3	9.5	0.3
COPD	12.8	12.1	0.6
Chronic renal failure	4.8	4.9	0.9
Congestive heart failure	15.8	15.9	0.96
Current smoker	13.6	14.6	0.4
Dementia	19.4	19.2	0.9
Diabetes mellitus	28.5	29.7	0.5

Disseminated cancer	1.5	1.5	0.99
Drug use disorder	5.5	5.8	0.2
Functionally dependent health status	35.8	33.8	0.3
History of myocardial infarction	1.6	1.9	0.6
Hypertension requiring medication	87.4	86.2	0.3
Liver disease	2.0	2.3	0.6
Major psychiatric illness	26.2	25.8	0.8
Obesity	2.9	2.9	0.99
Peripheral vascular disease	5.5	5.7	0.9
Steroid use	3.2	3.1	0.8
