# Supplementary material for: Association of Discontinuing Preinjury Beta-Adrenergic Blockade Medications With Mortality in Severe Blunt Traumatic Brian Injury
Source: Ann Surg Open. 2023 Aug 29;4(3):e324. doi: 10.1097/AS9.0000000000000324 (PMC10513140; doi:10.1097/AS9.0000000000000324)
Supplement: Supplementary file 2 [file as9-4-e324-s002.pdf]

Supplemental Table 2. Patient Characteristics at Baseline prior to Propensity Score Matching

Patient Characteristic	Cohort				*p*-value
	Pre BB = No	Pre BB = Yes	Pre BB = No	Pre BB = Yes	
	TBI BB = No	TBI BB = No	TBI BB = Yes	TBI BB = Yes	
Patients, N	9,300	1,601	1,399	2,853	
Age, mean (SD)	57.9 (22.3)	75.3 (13.4)	65.7 (19.8)	77.4 (11.9)	<0.001
Age, %					
16-25y	10.6	0.3	4.3	0.04	<0.001
26-45y	20.1	2.5	12.2	1.3	
46-65y	27.7	19.2	26.5	14.1	
66-75y	14.2	20.7	19.2	22.6	
>75y	27.4	57.3	37.8	62.0	
Male, %	64.0	57.2	62.9	54.6	<0.001
Race, %					
White	78.2	84.3	76.8	87.2	<0.001
Black	16.5	12.2	19.2	9.4	
Other	5.4	3.6	4.0	3.4	
Payment					
Commercial	36.3	17.8	30.5	14.9	<0.001
Medicaid	13.7	5.3	10.4	3.6	
Medicare	39.9	72.9	52.2	77.1	
Uninsured/Self pay	7.1	2.7	4.1	2.5	
Other	3.0	1.3	2.8	1.9	
Injury, %					
Fall	58.3	85.0	71.6	89.7	<0.001
MVC	16.9	8.0	11.3	5.5	
Motorcycle	6.7	1.6	4.4	0.8	

Pedal	7.1	1.9	5.1	1.1	
Struck	6.9	2.3	4.5	2.1	
Other	4.1	1.2	3.1	0.8	
Injury Severity Score, %					
5-15	32.6	36.1	30.6	40.0	<0.001
16-24	34.3	33.0	31.4	31.7	
25-35	27.3	28.4	32.5	27.1	
>35	5.8	2.5	5.5	1.2	
AIS Head/neck>2, %	100.0	100.0	100.0	100.0	0.99
AIS Chest>2, %	16.3	8.7	13.5	5.7	<0.001
AIS Abdomen>2, %	3.2	1.1	2.7	0.7	<0.001
AIS Extremity>2, %	7.8	4.7	6.6	3.3	<0.001
ED Heart Rate, %					
51-120, bpm	89.2	90.9	87.4	91.9	<0.001
> 120	7.1	4.3	8.5	3.0	
0-50	1.3	1.5	1.5	0.8	
Missing	2.4	3.3	2.6	4.3	
ED Systolic Blood Pressure, %					
> 90, mmHg	94.6	93.5	94.6	94.6	<0.001
61-90	2.3	2.5	2.0	0.9	
≤ 60	0.5	0.4	0.1	0.1	
Missing	2.6	3.6	3.3	4.4	
Glasgow Coma Scale Motor, %					
6	65.0	72.7	64.2	79.1	<0.001
5-2	16.0	11.9	16.4	7.4	
1	11.4	6.8	12.1	2.2	
Missing	7.6	8.6	7.3	11.3	
Pupil Response					

Both Reactive	76.7	76.5	76.1	74.7	<0.001
One Reactive	3.0	3.1	3.4	1.9	
Neither Reactive	5.0	4.1	5.1	1.3	
Unable to obtain or missing	15.3	16.3	15.4	22.1	
Midline Shift					
Yes	14.2	18.0	20.0	15.3	<0.001
No	81.9	79.3	74.6	81.4	
Not imaged	0.8	1.1	0.7	1.5	
Missing	3.1	1.6	4.7	1.8	
Transfer in, %	24.8	29.3	23.2	26.3	<0.001
Intubated, %	45.7	37.5	48.2	27.8	<0.001
Pre-arrival CPR	1.0	0.6	1.1	0.3	0.002
ICP Monitor	10.7	4.8	12.2	2.4	<0.001
Brain Operation	14.6	13.8	22.0	13.4	<0.001
Time to Intervention < 8hrs	12.5	9.1	16.7	6.3	<0.001
Comorbid diseases, %					
Active chemotherapy	0.8	0.9	0.8	1.2	0.3
Advanced directive	5.1	11.9	5.8	11.2	<0.001
Alcohol use disorder	15.3	10.6	16.9	8.8	<0.001
Angina	0.4	2.0	0.7	1.9	<0.001
Bleeding risk	12.9	45.2	22.3	49.4	<0.001
Cerebrovascular accident	3.6	8.4	4.8	9.3	<0.001
COPD	6.3	12.5	8.4	13.5	<0.001
Chronic renal failure	1.0	5.3	2.1	3.6	<0.001
Congestive heart failure	2.9	15.6	6.7	18.0	<0.001
Current smoker	26.6	15.1	21.2	10.7	<0.001
Dementia	10.9	18.5	11.4	20.3	<0.001
Diabetes mellitus	13.4	28.7	19.2	31.2	<0.001

Disseminated cancer	0.9	1.4	0.9	1.5	0.02
Drug use disorder	20.8	6.3	14.4	4.5	<0.001
Functionally dependent health status	18.2	34.2	21.2	43.6	<0.001
History of myocardial infarction	0.3	1.8	1.3	1.4	<0.001
Hypertension requiring medication	32.4	86.6	52.7	90.3	<0.001
Liver disease	1.5	2.2	0.9	1.5	0.04
Major psychiatric illness	24.1	26.1	23.6	29.6	<0.001
Obesity	1.9	3.0	2.8	2.1	0.01
Peripheral vascular disease	1.5	5.3	2.4	5.8	<0.001
Steroid use	1.7	3.2	2.0	3.3	<0.001

BB, beta blocker medication; TBI, traumatic brain injury; SD, standard deviation; y, year; MVC, motor vehicle crash; AIS, Abbreviated Injury Scale; ED, emergency department; bpm, beats per minute; mmHg, millimeters of mercury; ICP intracranial pressure monitor; hrs, hours; COPD, chronic obstructive pulmonary disease.
