# Supplementary material for: Association of Discontinuing Preinjury Beta-Adrenergic Blockade Medications With Mortality in Severe Blunt Traumatic Brian Injury
Source: Ann Surg Open. 2023 Aug 29;4(3):e324. doi: 10.1097/AS9.0000000000000324 (PMC10513140; doi:10.1097/AS9.0000000000000324)
Supplement: Supplementary file 3 [file as9-4-e324-s003.pdf]

Supplemental Table 3. Univariate (pre-matching) analysis and pairwise comparisons of treatment cohorts and mortality.

A. All TBI Patients

Outcome	Cohort				*p*-value
	Pre BB = No	Pre BB = Yes	Pre BB = No	Pre BB = Yes	
	TBI BB = No	TBI BB = No	TBI BB = Yes	TBI BB = Yes	
Patients, N	9,300	1,601	1,399	2,853	
Mortality, % (N)	7.8 (723) †	13.2 (212)*	8.3 (116) †	6.8 (193)*	<0.001
Mortality or Hospice, % (N)	10.8 (1,005)	21.1 (338)	12.6 (176)	12.5 (357)	<0.001

B. Isolated TBI Patients

Outcome	Cohort				*p*-value
	Pre BB = No	Pre BB = Yes	Pre BB = No	Pre BB = Yes	
	TBI BB = No	TBI BB = No	TBI BB = Yes	TBI BB = Yes	
Patients, N	7,190	1,400	1,156	2,589	
Mortality, % (N)	6.3 (450) €	12.6 (176) ¥	7.2 (83) €	6.6 (170) ¥	<0.001
Mortality or Hospice, % (N)	9.8 (704)	20.4 (286)	11.6 (134)	12.2 (316)	<0.001

Legend: Abbreviations: BB, beta blocker medication; TBI, traumatic brain injury

* Chi Squared test was conducted to further evaluate the pairwise comparison for Mortality of: Pre BB = Yes, TBI BB = No verses Pre BB = Yes, TBI BB = Yes, p value < 0.001

† Chi Squared test was conducted to further evaluate the pairwise comparison for Mortality of: Pre BB = No, TBI BB = No verses Pre BB = No, TBI BB = Yes, p value = 0.5

¥ Chi Squared test was conducted to further evaluate the pairwise comparison for Mortality of: Pre BB = Yes, isolated TBI BB = No verses Pre BB = Yes, isolated TBI BB = Yes, p value < 0.001

€ Chi Squared test was conducted to further evaluate the pairwise comparison for Mortality of: Pre BB = No, isolated TBI BB = No verses Pre BB = No, isolated TBI BB = Yes, p value = 0.2
