# Supplementary material for: Association of Discontinuing Preinjury Beta-Adrenergic Blockade Medications With Mortality in Severe Blunt Traumatic Brian Injury
Source: Ann Surg Open. 2023 Aug 29;4(3):e324. doi: 10.1097/AS9.0000000000000324 (PMC10513140; doi:10.1097/AS9.0000000000000324)
Supplement: Supplementary file 4 [file as9-4-e324-s004.pdf]

Supplemental Table 4. Propensity Score Matched analysis for each cohort of interest for all patients with a TBI, adjusted for hospital-level clustering

A. Cohort: Continuation of pre-injury BB verses patients that did not receive continuation of BB post-injury.

Outcome	Cohort		Odds Ratio	95% Confidence Interval	*p*-value
	Pre BB = Yes	Pre BB = Yes			
	TBI BB = No	TBI BB = Yes			
Patients, N	1,502	1,502	--	--	--
Mortality, % (N)	11.8 (177)	8.9 (133)	0.73	0.54-0.98	0.04
Mortality or Hospice, % (N)	19.4 (291)	14.0 (225)	0.73	0.55-0.98	0.04
Any Complication, % (N)	19.5 (293)	20.4 (307)	1.06	0.84-1.34	0.6
Serious Complication, % (N)	15.9 (238)	16.6 (250)	1.06	0.83-1.35	0.6
Cardiac Complication, % (N)	3.1 (46)	2.9 (43)	0.93	0.55-1.59	0.8
Cardiac Arrest, % (N)	1.4 (20)	1.0 (15)	0.71	0.36-1.40	0.3
Myocardial Infarction, % (N)	0.9 (14)	0.8 (12)	0.86	0.39-1.87	0.7
Cerebral Vascular Accident, % (N)	1.0 (15)	1.1 (16)	1.07	0.41-2.76	0.9

Payment; Commercial 16.4 vs. 19.4%, Medicaid 4.7 vs. 4.9%, Medicare 75.0 vs. 70.3%, Uninsured/Self pay 2.6 vs. 3.5%, Other 1.3 vs. 1.9%, *p*=0.04. No differences in all other covariates after propensity match.

B. Cohort: De novo initiation of BB verses patients that did not receive de novo initiation of BB.

Outcome	Cohort		Odds Ratio	95% Confidence Interval	*p*-value
	Pre BB = No	Pre BB = No			
	TBI BB = No	TBI BB = Yes			
Patients, N	1,387	1,387	--	--	--
Mortality, % (N)	9.7 (134)	8.2 (113)	0.83	0.64-1.08	0.2

Mortality or Hospice, % (N)	13.4 (186)	12.5 (173)	0.92	0.78-1.08	0.3
Any Complication, % (N)	22.9 (318)	29.5 (409)	1.41	1.11-1.78	0.005
Serious Complication, % (N)	19.0 (263)	25.0 (347)	1.43	1.14-1.78	0.002
Cardiac Complication, % (N)	2.0 (27)	3.3 (46)	1.73	1.08-2.77	0.02
Cardiac Arrest, % (N)	0.9 (13)	1.4 (19)	1.47	0.61-3.55	0.4
Myocardial Infarction, % (N)	0.3 (4)	0.9 (12)	3.02	1.01-9.04	0.048
Cerebral Vascular Accident, % (N)	0.8 (11)	1.3 (18)	1.64	0.83-3.27	0.2

No differences in covariates after propensity match.
