# Supplementary material for: Association of Discontinuing Preinjury Beta-Adrenergic Blockade Medications With Mortality in Severe Blunt Traumatic Brian Injury
Source: Ann Surg Open. 2023 Aug 29;4(3):e324. doi: 10.1097/AS9.0000000000000324 (PMC10513140; doi:10.1097/AS9.0000000000000324)
Supplement: Supplementary file 5 [file as9-4-e324-s005.pdf]

Supplemental Table 5. Propensity Score Matched analysis for each cohort of interest for patients with an isolated TBI, adjusted for hospital-level clustering

A. Cohort: Continuation of pre-injury BB verses patients that did not receive continuation of BB post-injury.

Outcome	Cohort		Odds Ratio	95% Confidence Interval	*p*-value
	Pre BB = Yes	Pre BB = Yes			
	TBI BB = No	TBI BB = Yes			
Patients, N	1,318	1,318	--	--	--
Mortality, % (N)	11.1 (146)	7.5 (99)	0.65	0.47-0.91	0.01
Mortality or Hospice, % (N)	18.6 (245)	14.3 (189)	0.73	0.54-0.999	0.05
Any Complication, % (N)	17.5 (230)	19.0 (251)	1.11	0.87-1.42	0.4
Serious Complication, % (N)	13.9 (183)	15.4 (203)	1.13	0.88-1.45	0.3
Cardiac Complication, % (N)	2.6 (34)	2.4 (31)	0.91	0.51-1.63	0.8
Cardiac Arrest, % (N)	0.9 (12)	0.8 (10)	0.83	0.34-2.04	0.7
Myocardial Infarction, % (N)	1.0 (13)	0.7 (9)	0.69	0.30-1.57	0.4
Cerebral Vascular Accident, % (N)	0.8 (11)	0.9 (12)	1.09	0.38-3.13	0.9

No differences in covariates after propensity match.

B. Cohort: De novo initiation of BB verses patients that did not receive de novo initiation of BB.

Outcome	Cohort		Odds Ratio	95% Confidence Interval	*p*-value
	Pre BB = No	Pre BB = No			
	TBI BB = No	TBI BB = Yes			
Patients, N	1,151	1,151	--	--	--
Mortality, % (N)	8.8 (101)	7.0 (81)	0.79	0.59-1.04	0.09

Mortality or Hospice, % (N)	13.0 (149)	11.5 (132)	0.87	0.74-1.03	0.1
Any Complication, % (N)	19.0 (219)	25.1 (289)	1.43	1.12-1.82	0.004
Serious Complication, % (N)	14.5 (167)	20.2 (233)	1.50	1.13-1.98	0.005
Cardiac Complication, % (N)	2.0 (23)	2.7 (31)	1.36	0.72-2.57	0.3
Cardiac Arrest, % (N)	1.0 (11)	0.8 (9)	0.82	0.30-2.20	0.7
Myocardial Infarction, % (N)	0.4 (4)	0.7 (8)	2.00	0.65-6.17	0.2
Cerebral Vascular Accident, % (N)	0.7 (8)	1.3 (15)	1.89	0.82-4.33	0.1

No differences in covariates after propensity match.
